# Hepatosplenic protective role of quercetin in gamma-irradiated rats: modulation of oxidative stress, inflammatory and TGF-β signaling pathways

**DOI:** 10.1186/s12906-026-05338-x

**Published:** 2026-04-01

**Authors:** Fatma Y. Abdou, Ola A. Gharib, Hanan A. Fahmy

**Affiliations:** 1 Department of Drug Radiation Research, National Center for Radiation Research and Technology, Egyptian Atomic Energy Authority (EAEA), Cairo, Egypt; 2 Pharmacology and Toxicology Lab., Department of Drug Radiation Research, National Center for Radiation Research and Technology, Egyptian Atomic Energy Authority (EAEA), Cairo, Egypt

**Keywords:** Quercetin, Hepatotoxicity, Gamma radiation, TGF-β, Radioprotective medication

## Abstract

**Purpose:**

This study evaluates quercetin’s ability to prevent liver and spleen disorders caused by gamma radiation in an experimental rat model and investigates the potential mechanism.

**Methods:**

Four sets of eight rats were randomly selected. Group I served as a negative control, and Group II received a single dose of γ-radiation (7 Gy). Group III was given 200 mg/kg of quercetin daily for ten days. Group IV: Rats were pretreated with quercetin for ten days; on the seventh day of quercetin treatment, the animals were subjected to a single dose of γ-radiation (7 Gy). AST, ALT, albumin and total protein levels are measured in serum as well as oxidative biomarkers contents (ROS and GSH) are estimated in liver and spleen tissues.IL-6 and TGF-β protein expression are quantified immunohistochemically in addition to histopathological examination of liver and spleen tissues of rats.

**Results:**

The liver and spleen tissues of irradiated rats have lower amounts of GSH and higher levels of reactive oxygen species (ROS) as compared to the control. Additionally, there was an increase in serum alanine transaminase (ALT) and aspartate aminotransferase (AST) and upregulation of transforming growth factor beta (TGF-β) and interleukin-6 (IL-6) expression, while serum albumin and total protein concentrations decreased. Conversely, oral quercetin pretreatment significantly improved indicators of liver function. Histopathological architecture and immunohistochemical analysis of fibrogenic and inflammatory biomarkers (IL-6 and TGF-β) validated the bioactivity of the flavonoid. By controlling IL-6 and TGF-β expression, quercetin reduced radiotherapy-induced liver damage, possibly via mitigating liver oxidative stress, inflammation, and fibrosis.

**Conclusion:**

To reduce the hepatic and splenic toxicity caused by radiation therapy, quercetin may be administered as a safe, stand-alone radioprotective medication.

**Graphical Abstract:**

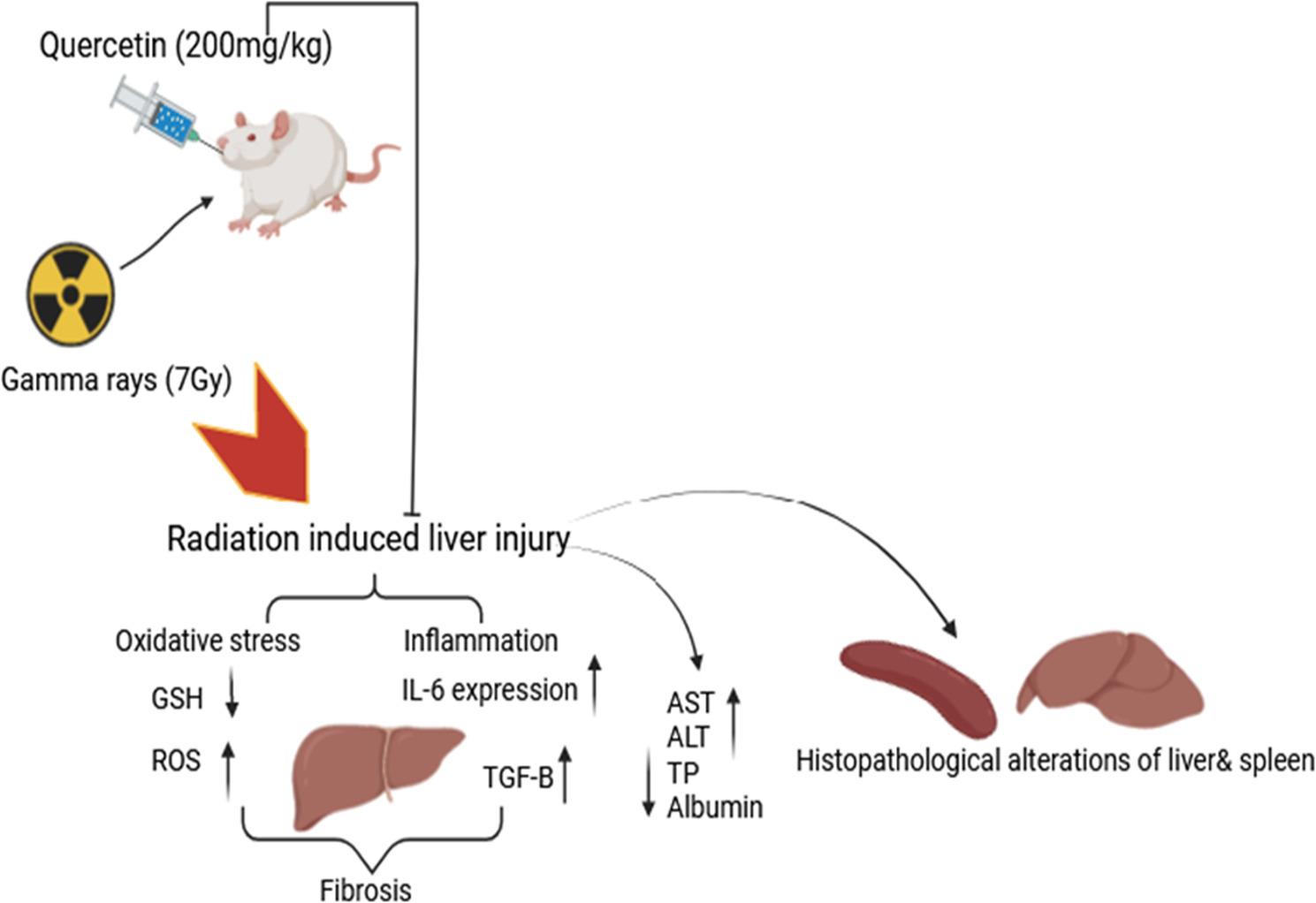

## Introduction

Although radiation is one of the primary ways often employed in cancer treatment, it inevitably reaches the area of treatment in healthy tissues and is negatively impacted by the acute and chronic side effects of radiation [1]. Living organisms are constantly subjected to oxidative stress and hazardous risks such as radiation, pollution, and poisons. Natural and manmade sources of ionizing radiation (IR) cause direct and indirect harm to cells [2]. Increased reactive oxygen species (ROS) produced by the stress situation of IR induce lipid, DNA, and protein damage [3].

Oxidative stress is involved in the pathophysiology of several diseases. Although cells naturally produce reactive oxygen species (ROS) to support metabolic processes, when ROS levels are high, cellular antioxidant defense mechanisms are overwhelmed, leading to a cascade of molecular damage [4]. The increased synthesis of reactive oxygen species (ROS) during radiation therapy brought on liver fibrosis [5]. Increased ROS production by activated inflammatory and immunological cells combating intruders is associated with inflammatory processes [6]. Continuous exposure to ROS generates oxidative stress, chronic inflammation, and diseases with oxidative stress and inflammation as common components [7]. Antioxidant treatments might provide an approach for avoiding radiation damage to normal tissues [8]. As a result, there is increased interest in examining the radio-protective potential of numerous natural chemicals in order to develop useful drugs that can protect against the detrimental effects of irradiation [9].

The hepatotoxic nature of medications, industrial pollutants, drug-induced hepatotoxicity, and gamma ray exposure have all been identified as key causes of liver disease [10]. Attempts have been undertaken to protect against the detrimental effects of ionizing radiation using pharmaceutical interventions [11]. Several hepatoprotective drugs, including natural substances such as bioactive compounds, have been shown to counteract ROS-mediated tissue damage through antioxidant and free radical scavenging activities [12].

Flavonoids are attracting great interest now for their potential pharmacological characteristics [13]. According to a number of in vitro studies, flavonoids can reduce apoptosis-mediated cell death, lower the generation of proinflammatory mediators, regulate the degree of oxidative stress, and have other beneficial effects [14]. Studies on animals have proven flavonoids’ anti-inflammatory, anticancer, neuroprotective, and other health-promoting properties [15]. Herbal extracts, such as quercetin, are widely distributed flavonoid compounds that have showed a range of pharmacological advantages [16, 17].

Quercetin (3,30,40,5,7-pentahydroxyflavone), a unique bioflavonoid, is found in high amounts in green vegetables, onions, fruits, and legumes [18]. Quercetin is a stronger antioxidant than other antioxidant substances, such as vitamin E, vitamin C, and b-carotene [19] besides its anti-inflammatory, and antifibrotic properties [20, 21]. Some animal models have verified quercetin’s beneficial effects on liver damage and fibrosis [22, 23]. Quercetin has been shown to block the Cytochrome P450 2E1 (CYP2E1) enzyme during diabetes development, preventing oxidative damage to the liver [24]. It is regarded a potent antioxidant flavonoid against reactive oxygen species, formed during normal oxygen metabolism or triggered by external damage; also, it has anti-inflammatory [25], vasodilatory [26], and angiogenic properties [27].

Quercetin has been shown to afford a beneficial effect in a variety of liver diseases [28]. However, the precise mechanisms by which quercetin protects the liver from injury must be identified. As a result, in the current investigation, we investigated how quercetin enhanced liver function besides liver and spleen tissue damage induced by gamma radiation, as well as the potential underlying mechanisms in rats through modulation of oxidative stress, inflammatory and TGF-β signaling pathways.

## Materials and methods

### Animals

Thirty-two adult male Wistar albino rats (150–180 g, 3 months old) were obtained from National Center for Radiation Research and Technology, Cairo, Egypt. The animals were acclimatized in the animal facilities of the National Center for Radiation Research and Technology (NCRRT), Egyptian Atomic Energy Authority (EAEA), Cairo, Egypt. Rats were kept in average humidity, temperature, and diet settings while being preserved, with unlimited access to water and a normal feed. The National Center for Radiation Research and Technology (NCRRT) Animal Care Committee, Egyptian Atomic Energy Authority, Cairo, Egypt, has approved the research ethics for animal care (Permit Number:7PA/ 24). The committee is organized and operates in accordance with the CIOMS and ICLAS International Guideline Principles for Biomedical Research Involving Animals, 2012. This study was reported in accordance with the ARRIVE standards.

### Chemicals

**Fig. 1:**
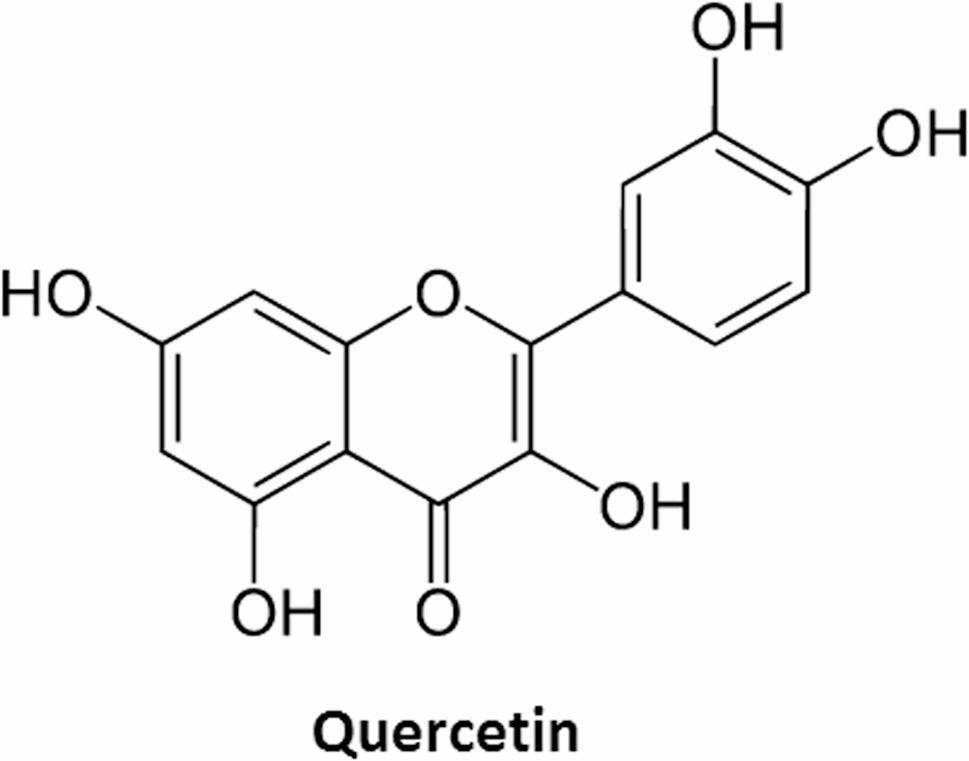
Chemical structure of Quercetin Flavonoid [29]

Highest purity grade of chemicals and solvents were used in all tests. Quercetin (purity ≥ 98%) Fig. 1 [29] and all other molecular grade chemicals were purchased from Sigma-Aldrich Pvt. Limited (USA).

### Irradiation to animals

Gamma cell-40 at the NCRRT (Cesium^137^, produced by the Atomic Energy of Canada Limited, Ontario, Canada) have been used to expose rats to 7 Gy as a single dose with a dose rate of 0.333 Gy/min for a total exposure time of 21 min [30, 31]. Cesium cell was standardized by alanine dosimetry relative to a primary standard. Correction was made daily for humidity, barometric pressure and temperature.

### Experimental design

Total number of thirty-two [32] rats, were randomly divided into four groups, (8 animals in each group). Group I: rats were received normal saline for ten days and served as a normal group (negative control). Group II: rats were exposed to a single dose of gamma radiation (7 Gy) at the 7th day. Group III: rats were received quercetin in a dose (200 mg/kg, daily for ten days p.o.). Group IV: rats were pretreated with quercetin for ten days; on the seventh day of quercetin treatment, the animals were subjected to a single dose of γ-radiation (7 Gy) as in group 2. Quercetin treatment was administered in a dose of 200 mg/kg as previously reported [32, 33]. Animals are typically fasted for 24 h before being sacrificed, drinking just water. The rats were sacrificed 24 h post the last dose of quercetin treatment.

At the end of the experiment, the rats were anesthetized with an intraperitoneal injection of thiopental (50 mg/kg). Blood was collected from the retro-orbital sinus under anesthesia using non-heparinized capillary tubes for serum separation. The rats were euthanized under anesthesia via cervical dislocation, and the liver and spleen tissues were removed, cleaned with saline, dried, and weighed. Each group underwent two sets of experiments: one for biochemical measurements and the other for histological and immunohistochemical comparisons of extracted tissue samples from all groups.

#### Sample collection and processing

Sera were frozen at -80 °C after being centrifuged at 3000 rpm for 15 min. Using a homogenizer (Glas-Col, USA), hepatic and spleen tissue samples were homogenized at 1:5 (weight: volume) (w: v) in phosphate buffer (pH 7.4). The supernatant separation was then completed by centrifugation at 10,000 rpm for 15 min (Cooling centrifuge, Hettich, MIKRO 22R, Germany), and the samples were stored at -80 °C for the analysis of oxidative stress biomarkers.

#### Assessment of enzyme markers of liver damage

Alanine aminotransferase (ALT) and aspartate aminotransferase (AST) were measured in serum using enzymatic colorimetric kits in accordance with the technique used by Reitman and Frankel [34], (Biodiagnostics, Egypt). Albumin and total protein levels were determined using the method utilized by Gornal et al., 1949 [35], Doumas et al. [36], respectively (Biodiagnostics, Egypt).

#### Assessment of oxidative stress biomarkers

Reduced glutathione (GSH) and Reactive Oxygen Species (ROS) were assessed in liver and spleen homogenates. Reduced glutathione (GSH) was determined according to Ellman’s method [37]. Where 0.5 ml of 10% TCA and 0.5 ml of homogenate, 1:5 (w: v), were placed in a centrifuge tube. The tube was centrifuged at 3000 for 10 min after being softly and sporadically shook for 15 min. A spectrophotometer (UNICAM 5625, UV/VIS, England) was used to measure the absorbance at 412 nm against a reagent blank within five minutes after aliquots of 0.5 ml of the resulting clear supernatant were combined with 1 ml of phosphate buffer (pH 8) in separate tubes and 0.1 ml of Ellman’s reagent was added. The findings were expressed in mmol of GSH/g of wet tissue. ROS generation was measured by the assay for intracellular conversion of nitro blue tetrazolium (NBT) to formazan by superoxide anion [38, 39]. The absorbance of the blank and the samples at 570 nm were measured spectrophotometrically and expressed as µmol NBT reduced /g tissue.

### Histopathological examinations for liver and spleen

Specimens of liver and spleen tissue were fixed in 10% formol saline, cut off, cleaned, and dehydrated using increasing alcohol grades. After being dehydrated, the specimens were sectioned at a thickness of 4–6 μm, embedded in paraffin blocks, and cleaned in xylene. For histological analysis using an electric light microscope, the acquired tissue portions were deparaffinized with xylol and stained with Hematoxylin and Eosin (H&E) [40]. As previously reported, the frequency and severity of liver lesions were evaluated semi-quantitatively [41] utilizing a scale where grade 0: represents no visible damage, grade I: represents hepatocyte swelling, grade II: represents hepatocyte ballooning, grade III: represents lipid droplets in hepatocytes, and grade IV: represents hepatocyte necrosis [42]. Histological grading of Peri-arteriolar lymphoid sheath as 0 = normal, 1 = minimal, 2 = mild, 3 = moderate, 4 = marked [43].

### Immunohistochemical analysis

Immunohistochemical staining was performed on 4-µm-thick sections of liver tissues that had been paraffin-embedded and formalin-fixed. TGF-β (rabbit polyclonal AB from Santa Cruz Biotechnology, Santa Cruz, CA, diluted at 1:100, in phosphate buffered saline (PBS)) and IL-6 (rabbit polyclonal AB from Santa Cruz Biotechnology, Santa Cruz, CA, USA) were immunohistochemically stained and scored in two distinct hepatic tissue regions (peri-portal and peri-venular areas), in compliance with Kasprzak et al., [44]. Reactivity was primarily determined by counting the amount of cytoplasmic nuclear staining in each specimen’s high power field. In six fields per section, the percentage of TGF-β and IL-6 immunoreactive region was calculated (X400). Negative (0), weak (+), moderate (++), and marked (+++) were the classifications for the reactivity [45].

### Statistical analysis

The sample size of 32 rats (*n* = 8/group) was divided equally among four groups using the G*Power software (Version 3.1.9, Düsseldorf, Germany). The Kolmogorov-Smirnov test and Bartlett’s test were employed to assess data normality and variance homogeneity, respectively. The data that matched the assumptions for parametric analysis were analyzed using one-way ANOVA and Tukey’s multiple comparisons test. The data are shown as the mean ± SEM. To analyze hepatic and spleen damage scores, the Kruskal-Wallis test was employed, followed by the post-hoc test (Dunn’s test), and the results were given as medians. Pearson’s correlation test, followed by linear regression analysis, was performed to investigate the relationships between the chosen measured parameters. The statistical analyses were performed with GraphPad Prism software (version 6.01). The significance criterion for all statistical tests is *p* < 0.05.

## Results

### Effects of pretreatment of quercetin on serum liver biomarkers in irradiated rats

**Fig. 2:**
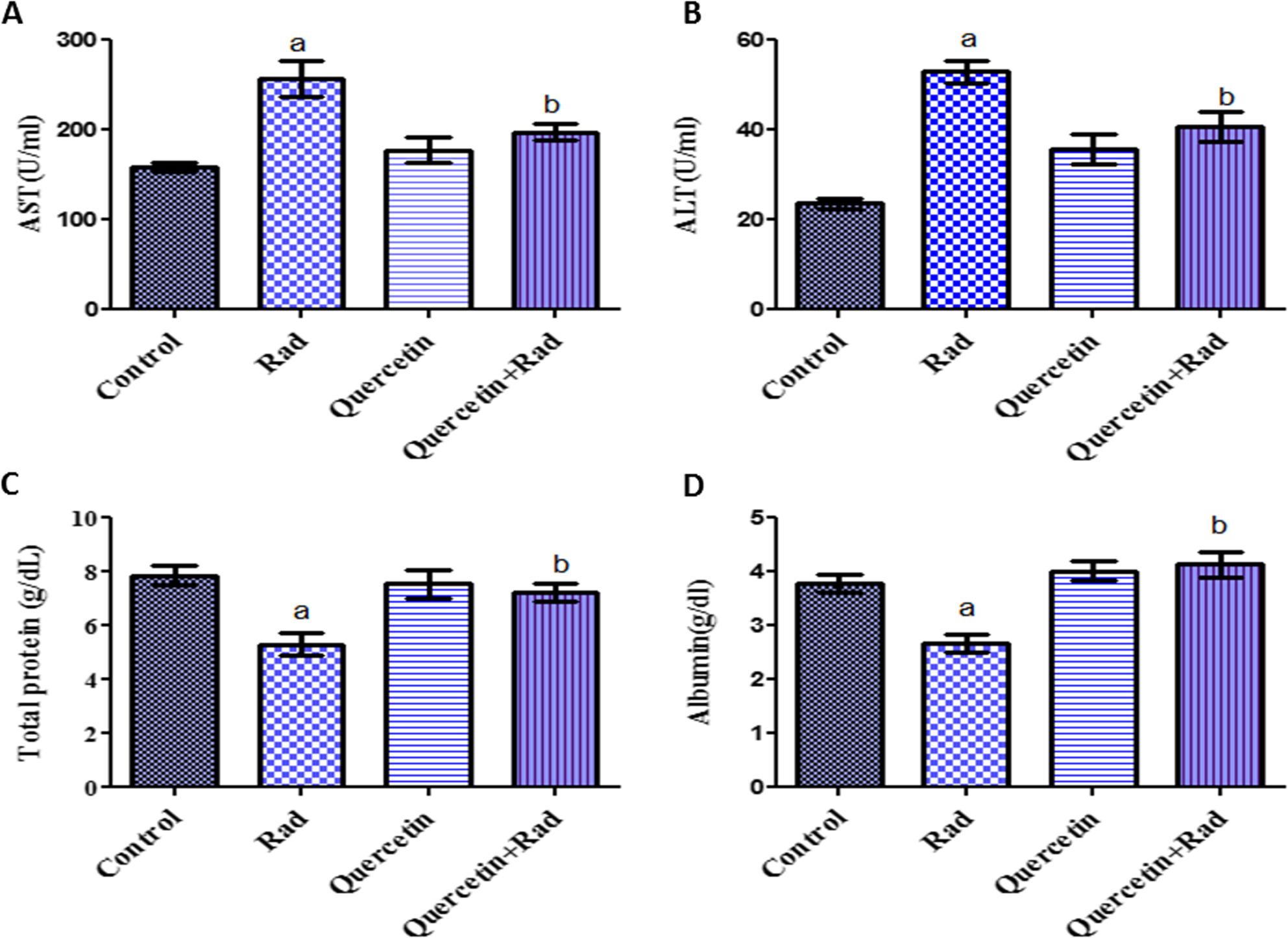
Effects of pretreatment of quercetin on serum liver biomarkers **A** AST, **B** ALT, **C** Total protein, and **D** Albumin levels in irradiated rats (7 Gy). Data presented as mean ± S.E of the mean. Statistical analysis was carried out by one-way ANOVA followed by Tukey-Kramer multiple comparisons test. a: significantly different from normal control group, b: significantly different from irradiated group. *p* < 0.05. (*n *= 8)

As evident in Fig. 2, gamma radiation exposure significantly increases rat serum liver ALT and AST with a decrease in serum albumin and total protein concentration as compared to control. Rats pretreated with quercetin for 10 days and subjected to gamma radiation (7 Gy) exhibited a significant improvement in all liver biomarkers compared to corresponding positive control group (*p* < 0.05). The present findings revealed that quercetin flavonoid ameliorated hepatic injury induced by exposure to high dose of gamma rays.

### Effects of pretreatment of quercetin on oxidative stress biomarkers in irradiated rats

**Fig. 3:**
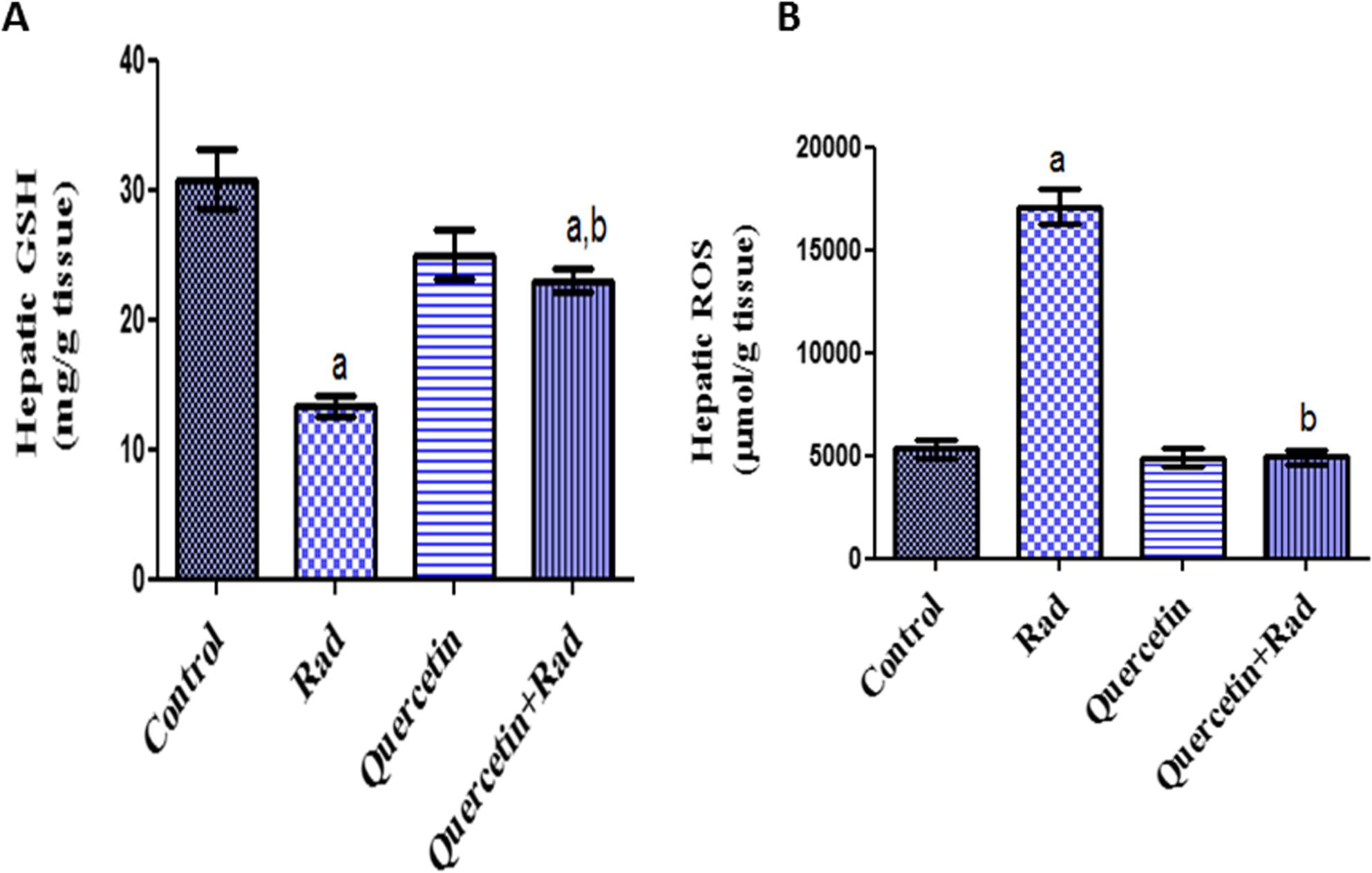
Effects of pretreatment of quercetin on hepatic (**A**) GSH and (**B**) ROS contents in irradiated rats (7 Gy). Data presented as mean ± S.E of the mean. Statistical analysis was carried out by one-way ANOVA followed by Tukey-Kramer multiple comparisons test. a: significantly different from normal control group, b: significantly different from irradiated group. *p* < 0.05. (*n* = 8)

**Fig. 4:**
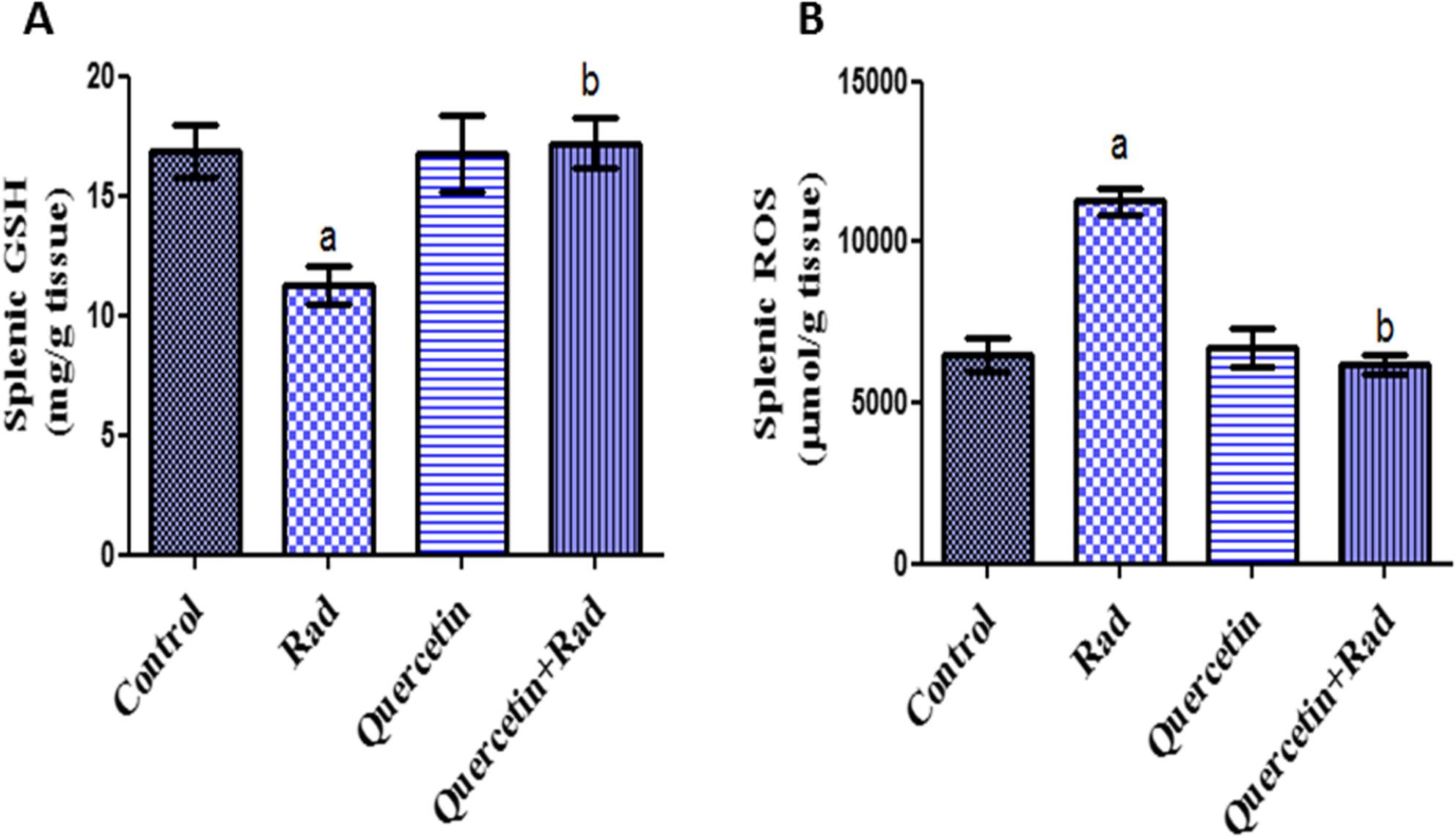
Effects of pretreatment of quercetin on splenic (**A**) GSH and (**B**) ROS contents in irradiated rats (7 Gy). Data presented as mean ± S.E of the mean. Statistical analysis was carried out by one-way ANOVA followed by Tukey-Kramer multiple comparisons test. a: significantly different from normal control group, b: significantly different from irradiated group. *p* < 0.05. (*n* = 8)

Oxidative stress parameters in liver and spleen homogenates are demonstrated in Figs. 3 and 4. In correlation to control values (*P* < 0.05), GSH activities were significantly decreased in rats exposed to gamma radiation in both tissues. When rats were exposed to gamma radiation, ROS levels were considerably greater in both organs than in normal control rats. Significant decrease in ROS and elevated levels of GSH in liver and spleen homogenates were noticed in irradiated rats pretreated with quercetin as equated to the irradiated group of rats. The obtained records showed that quercetin alleviated the oxidative stress-injuring effects induced by gamma radiation in hepatic and spleen tissues.

### Effects of pretreatment of quercetin on inflammatory marker (IL- 6) in irradiated rats

**Fig. 5:**
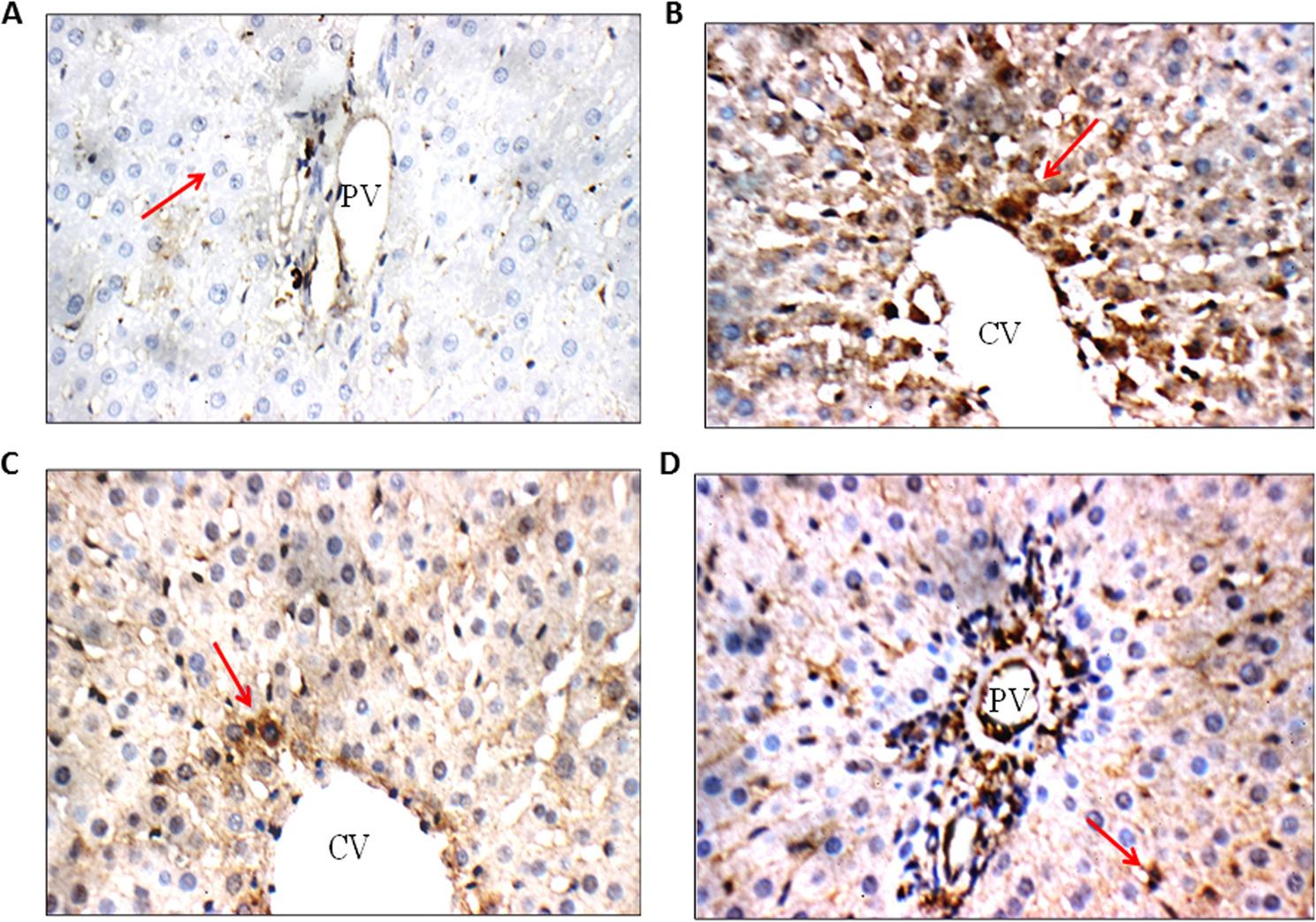
Photomicrographs of IL-6 immune-stained liver tissue sections showing: **A** Negative control group: showing negative cytoplasmic reactivity (0) for IL-6 in hepatocytes of peri-portal area (Red arrow) (IL-6 immunostaining X 400). **B** Radiation group (7Gy): showing marked cytoplasmic reactivity (+++) for IL-6 in hepatocytes of peri-portal area (Red arrow) (IL-6 immunostaining X 400) (**C**): Quercetin-treated group: showing weak cytoplasmic reactivity (+) for IL-6 in hepatocytes of peri-venular area (Red arrow) (**D**): Quercetin +Rad group: showing moderate cytoplasmic reactivity (++) for IL-6 in hepatocytes of peri-portal area (Red arrow) (IL-6 immunostaining X 400). Rad; radiation.

Both the staining intensity and the proportion of positive cells were taken into account when interpreting the data. The majority of the positive was cytoplasmic staining. Negative control group showed negative cytoplasmic reactivity (0) for IL-6 in hepatocytes of peri-portal and peri-venular areas. Moreover, radiation group (7 Gy) showed marked cytoplasmic reactivity (+++) for IL-6 in hepatocytes of peri-portal and peri-venular areas. Likewise, quercetin group showed weak cytoplasmic reactivity (+) for IL-6 in hepatocytes of peri-portal and peri-venular areas. Furthermore, the (quercetin + Rad) group showed moderate cytoplasmic reactivity (++) for IL-6 in hepatocytes of peri-portal and peri-venular areas, as shown in Fig. 5.

### Correlation between hepatic IL-6 and GSH & ROS

**Fig. 6:**
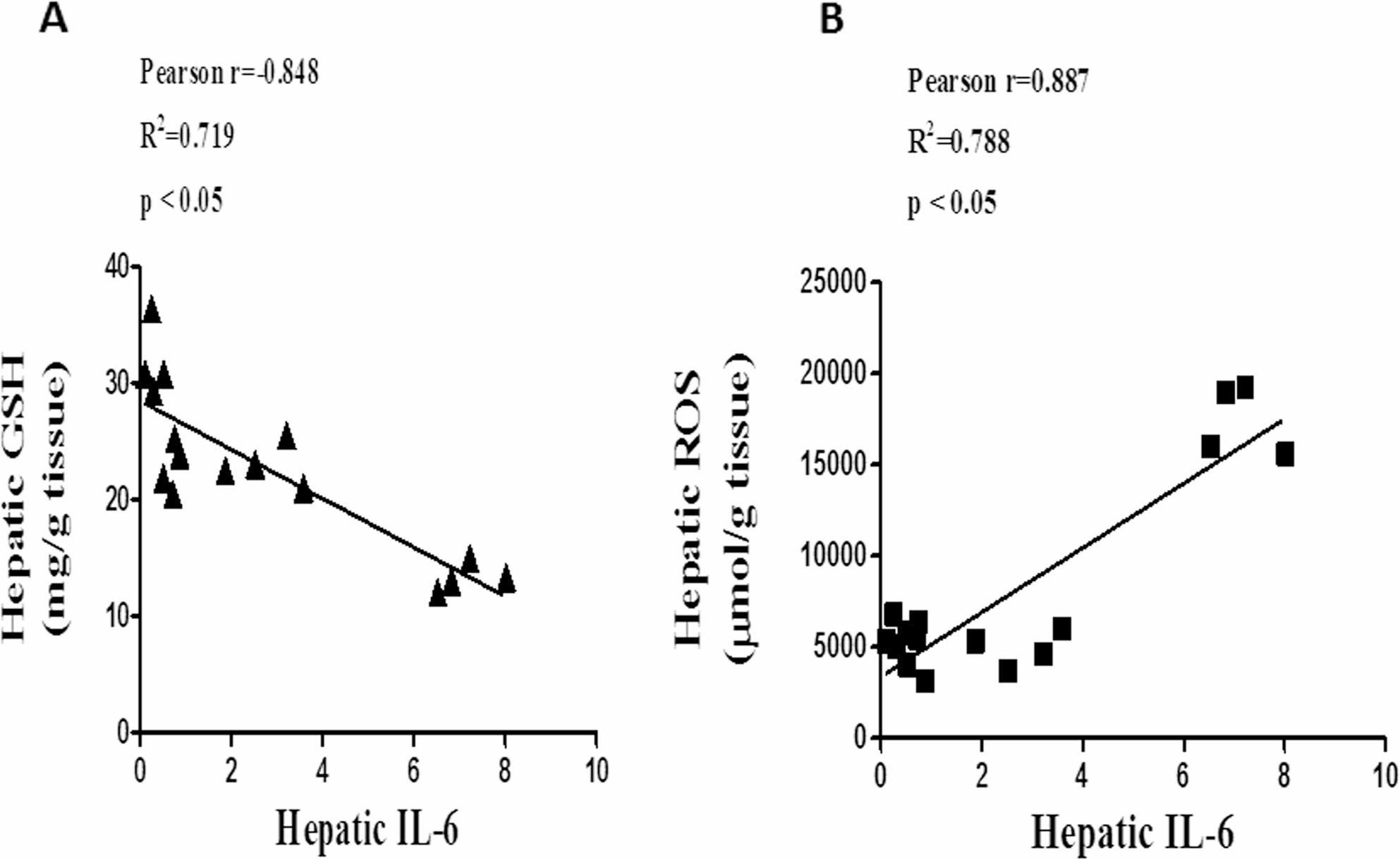
Correlation between hepatic expression of IL-6 and GSH & ROS. The expression of IL-6 protein is negatively and significantly correlated with (**A**) Hepatic GSH (r = -0.848, *P* <0.05). The expression of IL-6 protein is positively and significantly correlated with (**B**) Hepatic ROS (r = 0.887, *P* <0.05)

Figure 6 explores the relationship between IL-6 expression and GSH activities with ROS formation. Strong hepatic IL-6 expression in the Rad group (Fig. 5B) was linked to lower hepatic GSH and higher hepatic ROS (Fig. 3) in the current investigation. According to correlation analysis, IL-6 and hepatic ROS levels were positively and significantly correlated (Fig. 6B), while IL-6 and hepatic GSH levels were negatively and significantly correlated (Fig. 6A). Together, these results suggest that the anti-inflammatory and antioxidant qualities of quercetin may protect against tissue damage brought on by gamma radiation.

### Effects of pretreatment of quercetin on fibrogenic marker (TGF-β) in irradiated rats

**Fig. 7:**
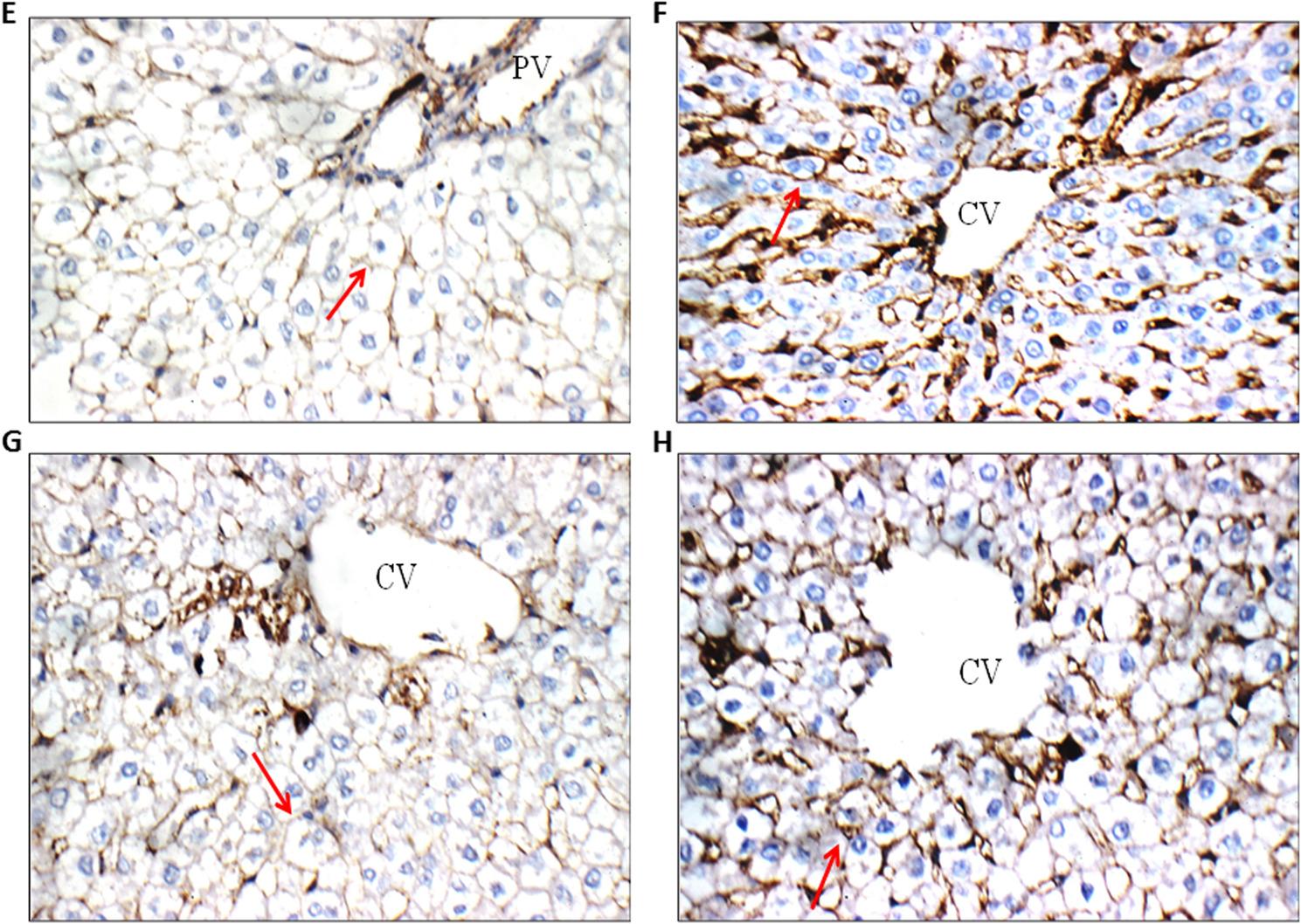
Photomicrographs of TGF-β immune-stained liver tissue sections showing: **E** Negative control group: showing weak cell membrane reactivity (+) for TGF-β in hepatocytes of peri-portal area (Red arrow) (TGF-β immunostaining X 400). **F** Radiation (7Gy) group: showing marked cell membrane reactivity (+++) for TGFβ in hepatocytes of peri-venular area (Red arrow) (TGF-β immunostaining X 400). **G** Quercetin-treated group: showing weak cell membrane reactivity (+) for TGF-β in hepatocytes of peri-venular area (Red arrow) (TGF-β immunostaining X 400). **H** Quercetin+ Rad group: showing moderate cell membrane reactivity (++) for TGF-β in hepatocytes of peri-venular area (Red arrow) (TGF-β immunostaining X 400). Rad; radiation.

In interpreting the results, the percentage of positive cells and the staining intensity were taken into account. It was thought that cell membrane staining was a positive sign. Negative control group showed weak cell membrane reactivity (+) for TGF-β in hepatocytes of peri-portal and peri-venular areas. Besides, radiation group showed marked cell membrane reactivity (+++) for TGF-β in hepatocytes of peri-portal and peri-venular areas. Quercetin group showed weak cell membrane reactivity (+) for TGF-β in hepatocytes of peri-portal and peri-venular areas. Moreover, (quercetin + Rad) group showed moderate cell membrane reactivity (++) for TGFβ in hepatocytes of peri-portal and peri-venular areas Fig. 7.

### Histopathology findings of liver tissues

**Fig. 8:**
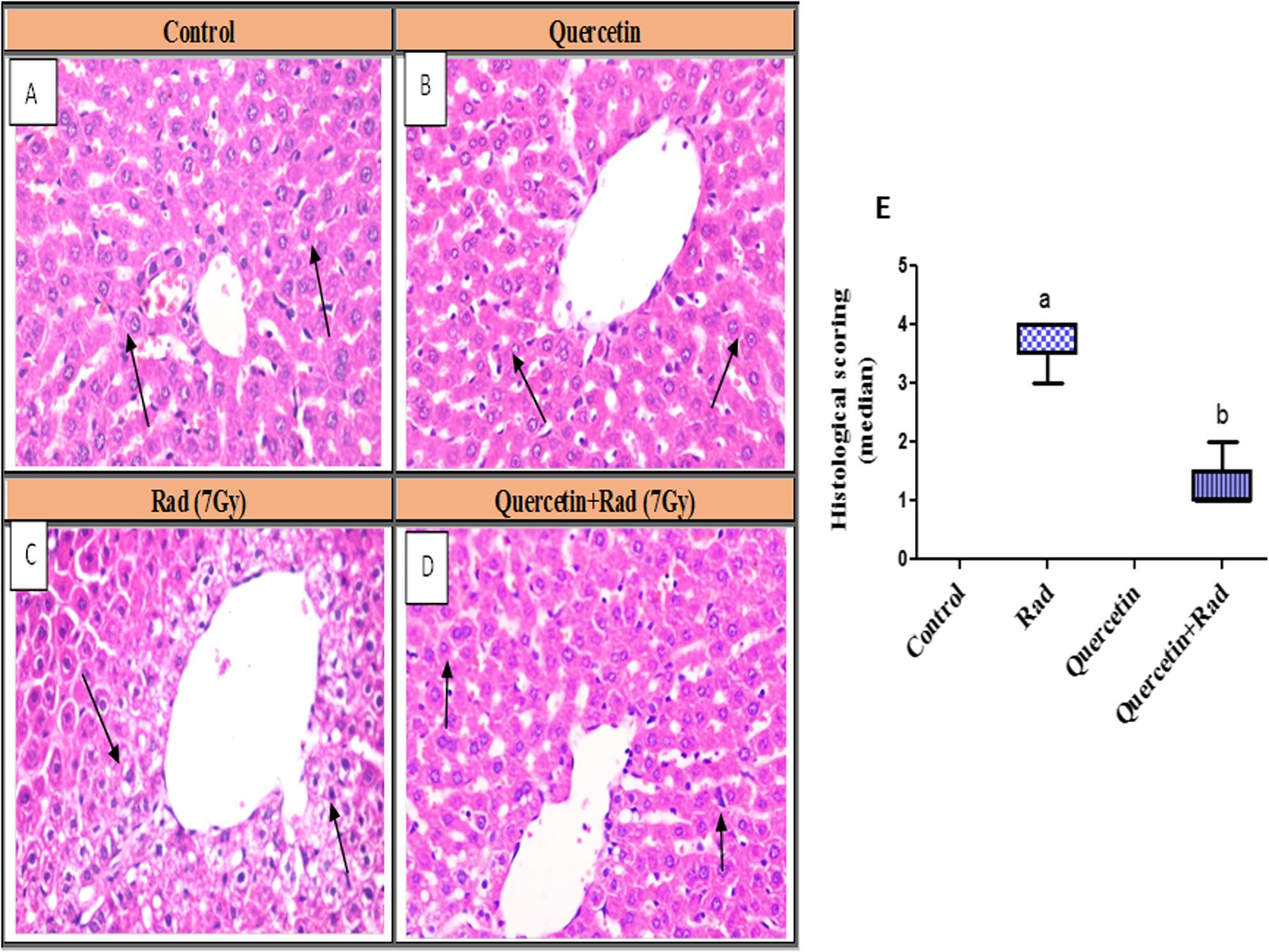
Photomicrograph of hepatic tissue section showing: **A** Control and **B** Quercetin-treated groups: showed normal histological structure of hepatic lobules (black arrows). **C** Rad (7Gy) group: revealed ballooning degeneration and apoptosis of hepatocytes (black arrows). **D** Quercetin+Rad group: demonstrated mild swelling of hepatocytes and hyperplasia of Kupffer cells (black arrows) (H&Ex400). **E** Histological scoring of hepatic damage. Each value represents the median (interquartile ranges) of three experiments. a: significantly different from normal control group, b: significantly different from irradiated group using Kruskal–Wallis test followed by post-hoc test (Dunn’s test). Rad: Radiation

Liver tissue section of control and quercetin, groups showed normal histological structure of hepatic lobules and organization of hepatic cords with prominent central hepatic vein. Polygonal hepatic cells were joined to one another in anastomosing plates, with borders that face either the sinusoids or adjacent hepatocytes (grade 0). Liver tissue section of animals group exposed to (7 Gy) revealed disorganization of hepatic cords and necrobiotic changes of hepatocytes. Vacuolation of hepatocytes around the terminal hepatic venules were detected. Pyknosis of hepatocytes nuclei were also seen. Apoptosis of hepatocytes which appeared as deeply eosinophilic apoptotic bodies contained nuclear fragments. Few numbers of intracellular fat droplets were noticed. Hyperplasia of Kupffer cells and segmental narrowing of hepatic sinusoids were observed (grade IV). On the other side, liver tissue section of animals group pretreated with quercetin then exposed to (7 Gy) gamma radiation, showed mild swelling of hepatocytes in the peripheral zone with central situated vesiculated nuclei with peripheral condensation of nuclear chromatin. Dilatation of hepatic sinusoids with hyperplasia of Kupffer cells were also noticed (grade I) Fig. 8. These outcomes showed that quercetin could lessen the histopathological architecture brought on by the administration of gamma radiation.

### Histopathology findings of spleen tissues

**Fig. 9:**
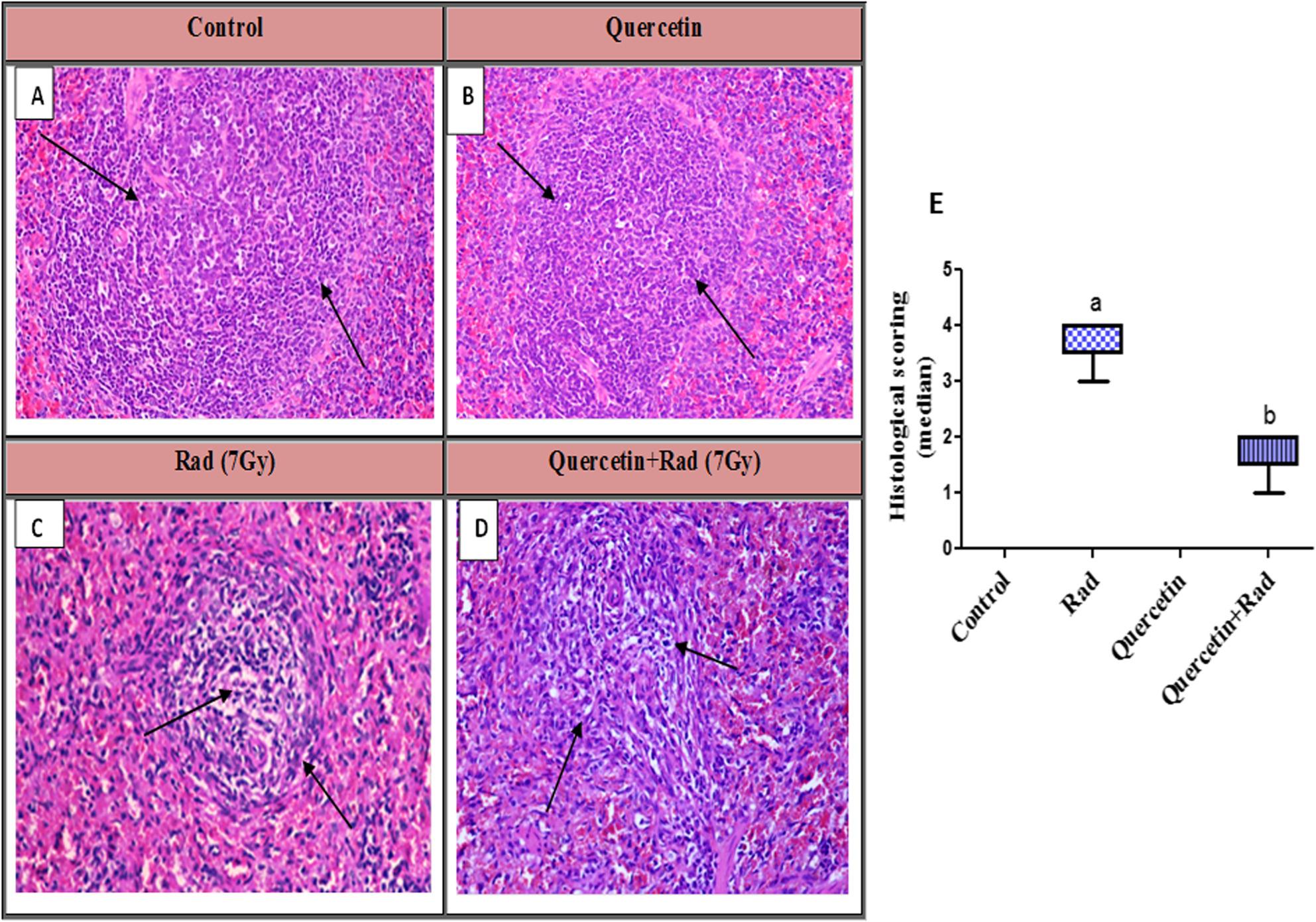
Photomicrograph of splenic tissue section showing: **A** Control and **B** Quercetin-treated groups: showed organization of periarteriolar lymphoid sheath floating in the red pulp with clear and prominent marginal area (black arrows). **C** Rad (7Gy) group: displayed marked depletion of white pulp and apoptosis of lymphocytes (black arrows). **D** Quercetin +Rad group: demonstrated mild depletion of white pulp with irregular periarteriolar lymphatic sheath (black arrows) (H&EX 200). **E** Histological scoring of spleen damage. Each value represents the median (interquartile ranges) of three experiments. a: significantly different from normal control group, b: significantly different from irradiated group using Kruskal–Wallis test followed by post-hoc test (Dunn’s test). Rad: Radiation

The transverse sections of control and quercetin groups, spleen exhibited numerous rounds, long or irregular aggregation of lymphoid cells called white pulp with organization of per arteriolar lymphoid sheath floating in the red pulp with clear and prominent margin grade (0). Spleen tissue section of animals group exposed to (7 Gy) gamma radiation, showed disorganization of splenic architecture and marked depletion of white pulp with marked reduction of lymphocytes number. Apoptosis of lymphocytes with thin per arteriolar lymphoid sheath were remarked. Expansion of red pulp and congestion of splenic sinuses with were also observed grade (IV). Conversely, spleen tissue section of animals group pretreated with quercetin then exposed to 7 Gy gamma radiation, revealed disorganization of splenic architecture and mild depletion of white pulp with reduction of lymphocytes number in periarteriolar lymphatic sheath with regular arrangement of red pulp grade (II) Fig. 9. Quercetin was able to reduce the histopathological architecture caused by gamma rays, as the current results showed.

## Discussion

Ionizing radiation induces the production of intracellular reactive oxygen species and pro-inflammatory cytokines, leading to hepatic damage [46]. Numerous hepatoprotective lead compounds have been created from natural resources [47]. Therefore, we postulated that pre-treatment with bioflavonoid antioxidant could be a beneficial tactic to prevent radiation injuring and reduce pathological changes [48].

Compared to normal control rats, the group of gamma-irradiated rats (7 Gy) in the current study showed a substantial drop in serum total protein and albumin levels and a significant increase in serum levels of liver function enzymes (AST & ALT) (*p* < 0.05). Furthermore, gamma radiation exposure significantly raised ROS levels in liver and spleen tissues and significantly decreased GSH levels. Moreover, hepatocytes in the peri-portal and peri-venular regions of the gamma irradiated group exhibited a notable cytoplasmic reactivity (+++) for IL-6. Likewise, hepatocytes in the peri-portal and peri-venular regions of the radiation group had a significant cell membrane reactivity (+++) for TGF-β. A liver tissue section from an animal group subjected to 7 Gy showed hepatocyte necrobiotic alterations and hepatic cord disarray. There was evidence of hepatocyte vacuolation surrounding the terminal hepatic venules. The nuclei of hepatocytes also showed signs of pyknosis. Nuclear fragments were seen in the profoundly eosinophilic apoptotic masses that resulted from hepatocyte apoptosis. Additionally, spleen tissue sample from a group of animals exposed to gamma radiation (7 Gy), revealed splenic architectural disarray, a significant decrease in the number of lymphocytes, and a considerable depletion of white pulp.

The current outcomes are in consistent with previous studies that revealed that gamma-rays-induced hepatic injury attained reduction in cellular antioxidant defense mechanisms; GSH content, and GPx, CAT and SOD enzymes activity [10, 49]. Increased formation of free radicals can target various cell components, causing metabolic alterations and macromolecule modifications that lead to oxidative stress brought on by ionizing radiation [50]. Moreover, radiation causes direct harm to hepatocytes through oxidative stress and an inflammatory reaction as reported previously [51]. By stimulating liver Kupffer cells and drawing circulating immune cells to infiltrate and activate, it then makes liver damage worse [52]. Hepatic stellate cells (HSC) and non-parenchymal cells (NPCs) participate in the process of liver fibrosis, which is primarily driven by TGF-β1, in the late stages of radiation-induced liver injury (RILI) [53]. Furthermore, it was reported that RILI causes the death of hepatocytes and eventually results in liver fibrosis [46]. It was previously stated that TGF-β1 expression is significantly increased in the liver following radiation and the development of fibrosis [53, 54].

Likewise, high-dose radiation causes hepatocyte necrosis and apoptosis, which in turn triggers the liver’s inflammatory response through a variety of routes [55, 56]. By activating multiple vital pathways, the interaction between damaged hepatocytes and liver NPCs accelerates the development of inflammation and liver fibrosis [57, 58]. A range of inflammatory mediators, including TNF-α, IL-6, IL-1β, PGE2 and ROS are subsequently produced and released by the activated Kupffer cells [59]. Meanwhile, the spleen, as an important component of the immune system, is responsible for blood cell production, blood filtering, the elimination of old blood cells, and infection resistance [60]. The resistance of cells and tissues to ionizing radiation is influenced by antioxidant systems and adaptive response capability. The spleen is among the most radiation-sensitive organs [61].

According to our findings, pretreatment with quercetin (200 mg/kg) for 10 days and subjected to gamma radiation (7 Gy) exhibited a significant improvement in all liver biomarkers compared to corresponding positive control group through several mechanisms involving antioxidative. Moreover, significant decrease in ROS and elevated levels of GSH in liver and spleen homogenates were noticed in irradiated rats pretreated with quercetin. Besides, the (quercetin + Rad) group showed moderate cytoplasmic reactivity (++) for IL-6 in hepatocytes of peri-portal and peri-venular areas. Likewise, the irradiated rats pretreated with quercetin, showed moderate cell membrane reactivity (++) for TGFβ in hepatocytes of peri-portal and peri-venular areas. Furthermore, concerning histopathological structure of hepatic and splenic tissues, quercetin mitigates the histopathological architecture in gamma irradiated rats.

The current results are similarly consistent with previous studies showed that quercetin may lower serum total bilirubin, ALP, ALT, and AST while improving liver lesions in some liver injury models [62, 63]. Other earlier study has revealed that quercetin has a hepatoprotective impact against liver disorders, which is believed to be due to its antioxidant properties, which are mostly determined by its chemical structure (28). Also, the antioxidant property was validated by quercetin treatment, which was in line with prior in vivo investigations [64, 65]. Moreover, quercetin’s anti-inflammatory and antioxidant properties can prevent diabetes and have hepatoprotective effects [66]. Additionally, serum transaminases and liver oxidative stress biomarkers were all shown to be significantly (*P* < 0.05) reduced by quercetin, which was said to have hepatoprotective efficacy [67]. Because of its antioxidant qualities, quercetin is thought to reduce malondialdehyde (MDA) levels while increasing glutathione (GSH) concentration. It does this by acting as a free radical scavenger, decreasing oxidative stress, and raising endogenous antioxidant levels [68]. This can help protect cells from damage caused by ROS [69].

Additionally, quercetin reduced the hepatic tissue damage brought on by Lead (Pb) and Al_2_O_3_ NPs and enhanced the hepatic antioxidant capacity [70]. Previous study showed that by blocking ferroptosis via IL-6/STAT3 signaling pathway, quercetin may mitigate this liver damage [71]. Quercetin therapy has been shown to successfully decrease liver pro-oxidant and anti-inflammatory markers in numerous animal models of liver injury, implying that quercetin may have considerable anti-inflammatory capabilities, particularly through scavenging actions [72].

Moreover, it was reported that quercetin controlled markers of hepatic stellate cell (HSC) activation and inflammation, including TNF-α, IL-6, IL-1β, COX-2, TGF-β, α-SMA, Colla1, Colla2, TIMP-1, MMP-1, and desmin [73]. In earlier work [74], quercetin treatment significantly reduced liver levels of TGF-β1 implying an evident antifibrotic effect. Hence, treatment with quercetin may reduce TGF-β1 levels in the liver, perhaps improving liver function and reducing fibrosis [23]. Concerning the histopathological alterations, quercetin likely alleviated histological abnormalities in the diabetic rats’ livers due to its primary antioxidant characteristics and its probable function in lowering oxidative stress and damage, as well as guarding against lipid and protein damage [75, 76]. Besides, It is previously reported that quercetin alleviates all histological markers of inflammation in liver tissues [28, 77]. In line with our findings, quercetin was found to increase antioxidant status and reduce oxidative damage in the spleens of rotenone-treated mice [78]. The significant antioxidant action of quercetin bioflavonoid was linked to the hydroxyl group on the side phenyl ring, which allows it to scavenge free radicals while also improving enzymatic antioxidants and glutathione [79, 80].

## Conclusion

The study demonstrated that the antioxidant flavonoid quercetin protects against oxidative stress and cellular damage in the liver and spleen caused by gamma radiation in rats. Key findings included improved liver function, reduced oxidative stress, and enhancement of antioxidant defense, along with decreased levels of inflammatory (IL-6) and fibrogenic (TGF-β) markers. Quercetin appears to alleviate radiation-induced liver damage through its antioxidant effects and modulation of inflammatory pathways, positioning it as a valuable candidate for further research in radiation injury protection and chronic liver disease prevention. Continued research is essential to better understand the mechanisms behind radiation-induced liver injury (RILI), which will aid in advancing targeted radiotherapy. This understanding is crucial for improving the quality of treatment and developing affordable targeted medications that feature lower toxicity and higher efficacy. Additionally, identifying further potential biomarkers for RILI is necessary.

## Data Availability

The datasets used and/or analyzed in the current investigation are available from the corresponding author upon reasonable request.

## Abbreviations

γ-radiation: Gamma radiation
ROS: Reactive oxygen species
ALT: Alanine transaminase
AST: Aspartate aminotransferase
TGF-β: Transforming growth factor beta
IL-6: Interleukin-6
CYP2E1: Cytochrome P450 2E
TNF-α: Tumor necrosis factor-alpha
GSH: Reduced glutathione
GPx: Glutathione peroxidase
CAT: Catalase
SOD: Superoxide dismutase
HSC: Hepatic stellate cells
NPCs: Non-parenchymal cells
RILI: Radiation-induced liver injury
PGE2: Prostaglandin E2
ALP: Alkaline Phosphatase
MDA: Malondialdehyde
PI3K: Phosphoinositide 3-Kinase
STAT3: Signal Transducer and Activator of Transcription 3
COX-2: Cyclooxygenase-2
Colla1: Collagen Type I Alpha
Colla2: Collagen Type I Alpha 2
TIMP-1: Tissue Inhibitor of Metalloproteinase-1
MMP-1: Matrix Metalloproteinase-1
LPS: Lipopolysaccharide
